# Target delineation and dose prescription of adaptive replanning intensity-modulated radiotherapy for nasopharyngeal carcinoma

**DOI:** 10.1186/s40880-019-0364-x

**Published:** 2019-04-15

**Authors:** Dehuan Xie, Wanqin Cheng, Shaowen Lv, Rui Zhong, Lei Wang, Jiang Hu, Mingli Wang, Shaomin Huang, Yong Su, Yunfei Xia

**Affiliations:** 1 Department of Radiation Oncology, Sun Yat-Sen University Cancer Center, State Key Laboratory of Oncology in Southern China, Collaborative Innovation Center for Cancer Medicine, 651 Dong Feng Road East, Guangzhou, 510060 Guangdong P. R. China; 2 Department of Oncology, Shunde Hospital of Southern Medical University, Foshan, 528399 Guangdong P. R. China; 3 Department of Radiation Oncology, Hainan General Hospital, Haikou, 570100 Hainan P. R. China; 4 Department of Radiation Oncology, National Cancer Center/Cancer Hospital & Shenzhen Hospital, Chinese Academy of Medical Sciences and Peking Union Medical College, Shenzhen, 518116 Guangdong P. R. China

Dear Editor,

Intensity-modulated radiotherapy (IMRT) has a distinct advantage of high conformity and is an appropriate technique for treating nasopharyngeal carcinoma (NPC). Previous studies have demonstrated that anatomical changes in the external contour, shape, and location of the target and critical structures are significant and result in dosimetric changes [1, 2]. Patients’ quality of life and clinical outcomes might be improved by IMRT replanning [3]. Therefore, replanning strategies should be considered instead of single-planning strategies throughout the entire course of radiotherapy. However, there are substantial controversies on (1) the appropriate time for target redelineation, (2) how to modify the target volumes, and (3) how to evaluate the modified plans. The present study provides a new perspective in replanning with regard to these three aspects.

We analyzed the data of 54 patients with newly diagnosed NPC between October 2013 and June 2016. The mean age was 45.5 years (range 18–67 years). All patients had undifferentiated non-keratinized carcinoma. According to the 7th edition of the Union for International Cancer Control (UICC)/American Joint Committee on Cancer (AJCC) staging system, 6 (11.1%), 17 (31.5%), 29 (53.7%), and 2 (3.7%) patients had stage IVb, IVa, III, and II diseases, respectively. Twenty-four (44.4%) patients received induction chemotherapy and concurrent chemoradiotherapy, 29 (53.7%) received concurrent chemoradiotherapy, and 1 (1.9%) received radiotherapy alone. The median duration of radiotherapy was 47 days (range 41–71 days). The median duration of interruption between two plans was 2 days (range 1–24 days). The baseline clinical characteristics are shown in Table 1.

**Table 1:** Baseline clinical characteristics of 54 patients with nasopharyngeal carcinoma

Characteristics	No of patients [cases (%)]
Sex	
Female	14 (25.9)
Male	40 (74.1)
Treatment	
Induction and concurrent chemoradiotherapy	24 (44.4)
Concurrent chemoradiotherapy	29 (53.7)
Radiotherapy alone	1 (1.9)
Staging (AJCC/UICC 2010)	
T stage	
T4	14 (25.9)
T3	24 (44.4)
T2	9 (16.7)
T1	7 (13.0)
N stage	
N3b	2 (3.7)
N3a	6 (11.1)
N2	34 (62.9)
N1	11 (20.4)
N0	1 (1.9)
M stage	
M0	54 (100)
M1	0 (0)
TNM stage	
IVb	6 (11.1)
IVa	17 (31.5)
III	29 (53.7)
II	2 (3.7)

*UICC* the Union for International Cancer Control, *AJCC* the American Joint Committee on Cancer

In plan-I radiotherapy, computed tomography simulation (CT-I) was performed for target delineation. The gross target volume of the nasopharynx (GTVnx)-I was defined as all gross lesions determined with clinical and imaging examinations. The high-risk clinical target volume (CTV1-I) was delineated with a 1-cm margin surrounding the GTVnx-I area. The low-risk clinical target volume (CTV2-I) was delineated with a 0.5-cm margin surrounding the CTV1-I. Any metastatic retropharyngeal lymph nodes and cervical lymph nodes were delineated as GTVrpn-I and GTVnd-I [4]. CTVrpn1-I and CTVnd1-I were delineated with a 0.5- and 1.0-cm expansion from the GTVrpn-I and GTVnd-I. CTVrpn2-I and CTVnd2-I were delineated with a 0.5-cm margin surrounding CTVrpn1 and CTVnd1-I, which included the bilateral prophylactically irradiated lymphatic drainage areas. In patients undergoing induction chemotherapy, target volumes were delineated according to the tumor appearance after induction chemotherapy [5].

On the basis of results of previous studies [1, 2], we chose to perform a second CT simulation (CT-II) after the 22nd fraction of radiotherapy, leaving 3 days for radiophysicists to make plan-II. For the first 11 patients, considering the inadequate time for determining adaptive plans, we implemented the plan-II radiotherapy after the 26th fraction. For the following patients, we implemented the plan-II radiotherapy after the 25th fraction. In plan-II radiotherapy, GTVnx/rpn/nd-II was defined as all residual diseases; CTV1/rpn1/nd1-II was the same as CTV1/rpn1/nd1-I; and CTV2/rpn2/nd2-II was not delineated (Fig. 1).

**Fig. 1:**
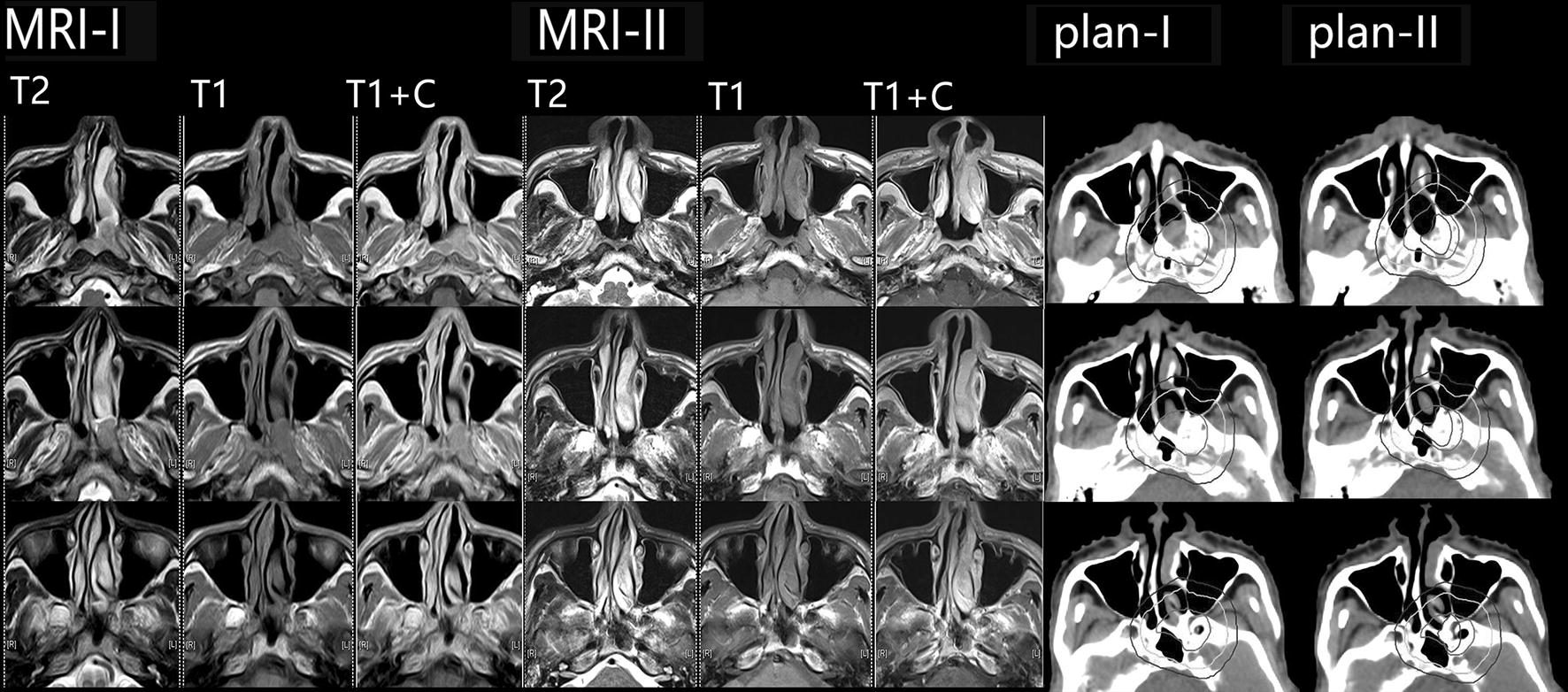
Illustration of target delineation in plan-I and plan-II. Magnetic resonance imaging (MRI)-I acquired before plan-I radiotherapy with cross-sectional T2-weighted images, T1-weighted images, and contrast-enhanced T1-weighted (T1 + C) images shows the primary tumor, which locates on the left side of the upper wall and extends into the nasal cavity, left medial pterygoid plate, and navicular fossa. In plan-I radiotherapy, the gross target volume of primary tumor (GTVnx-I) was outlined (red line). Clinical target volume 1-I (CTV1-I) (green line) is delineated with a 1.0-cm margin surrounding GTVnx-I. Clinical target volume 2-I (CTV2-I) (blue line) is delineated with a 0.5-cm margin surrounding CTV1-I. MRI-II acquired after 22 fractions of irradiation shows that the tumor greatly regressed. In plan-II radiotherapy, the residual tumor is delineated as GTVnx-II (red line). The regressing areas of the intracavitary area, the left medial pterygoid plate, and the navicular fossa lesion are included not in GTVnx-II but in CTV1-II (green line), which maintains the same as CTV1-I. CTV2-II is not delineated. GTVnx-I was copied to the CT-II for comparison (purple line)

A 3- to 5-mm margin surrounding the above targets was required for the delineation of the planning target volumes (PGTVnx, PGTVrpn, PGTVnd, PCTV1, and PCTV2).

Before June 2014, the doses prescribed were as follows: GTVnx/rpn/nd-I, 57 Gy in 26 fractions at 2.19 Gy/fraction; PCTV1/rpn1/nd1-I, 50 Gy in 26 fractions at 1.92 Gy/fraction; PCTV2/rpn2/nd2-I, 46–47 Gy in 26 fractions at 1.77–1.81 Gy/fraction; PGTVnx/rpn/nd-II, 11 Gy in 5 fractions at 2.2 Gy/fraction; PCTV1/rpn1/nd1-II, 10 Gy in 5 fractions at 2.0 Gy/fraction.

After June 2014, the doses prescribed were as follows: PGTVnx/rpn/nd-I, 53–54 Gy in 25 fractions at 2.12–2.16 Gy/fraction; PCTV1/rpn1/nd1-I, 47.5 Gy in 25 fractions at 1.90 Gy/fraction; PCTV2/rpn2/nd2-I, 45 Gy in 25 fractions at 1.8 Gy/fraction; PGTVnx/rpn/nd-II, 15–15.5 Gy in 7 fractions at 2.14–2.21 Gy/fraction; PCTV1/rpn1/nd1-II, 13.5 Gy in 7 fractions at 1.93 Gy/fraction.

The target delineation and dose prescription of organs at risk (OARs), including the brain stem, spinal cord, and optic chiasm, were performed according to Radiation Therapy Oncology Group (RTOG) 0225 protocol [4]. Under the dose tolerance limit requirements of the RTOG 0225 protocol [4], the dose constraints for OARs were calculated via multiplying the dose tolerance limit (D_tolerance limit_) by the percentage of dose of each plan in total dose.

The average weights of the patients were 61.2 ± 9.3 kg before radiotherapy and 58.2 ± 9.0 kg after the 22nd fraction of irradiation, without significant weight reduction (*P *> 0.05). GTVnx, GTVnd-R, volumes of bilateral parotids, and volumes of bilateral submandibular glands showed significant reductions after 22 fractions of irradiation (all *P* < 0.05), whereas other volume changes were not significant (Additional file 1: Table S1).

Nearly 100% of PGTV was irradiated with 95% of the prescription dose of PGTVnx in the two plans. No significant differences in the percentage of the mean dose (Dmean) in the total dose (Dmean%) of PGTVnx, bilateral PGTVrpn, or bilateral PGTVnd were observed between the two plans. Among the evaluated OARs, Dmean% values of the brain stem, spinal cord, optic chiasm, pituitary, oral cavity, oropharynx, hypopharynx, and thyroid gland were significantly different between plan-I and plan-II (*P *< 0.05) (Additional file 1: Table S2).

Adverse events were evaluated based on RTOG acute radiation morbidity scoring criteria. Grade 1–2 adverse events were mainly observed in the skin, oral mucosa, and salivary glands, whereas grade 0 adverse events were mainly observed in the hypopharynx mucosa and larynx mucosa. Grade 3 leukopenia, neutropenia, and thrombocytopenia were observed in 18 (33.30%), 13 (24.07%), and 5 (9.25%) patients, respectively; 1 (1.85%) developed grade 4 neutropenia.

The median follow-up period was 30 months (range 3–44 months). Three patients developed distant metastasis, and 4 developed locoregional failure, but none occurred in the regression area. The 3-year overall survival, local recurrence-free survival, and distant metastasis-free survival rates were 93.3%, 90.5%, and 91.4%, respectively.

Few studies have described the target redelineation in detail for replanning or modified dose prescription for tumor regression areas. Hansen et al. [6] used the same GTV in plan-II without extending it beyond the skin contour or into adjacent normal structures. Chitapanarux et al. [7] recontoured the GTV-II by removing the air cavity formed due to tumor shrinkage while maintaining the other dimensions of GTV-I. CTV-II was adapted by excluding the air cavity and noninvolved tissues. According to basic research and the results of definitive irradiation for NPC [8], a dose of 60 Gy delivered to subclinical lesions achieved good treatment efficacy. In the present study, upon disappearance/dissolution of tumor areas, the initial location of the tumor were included in CTV1-II, and the total dose delivered to the disappeared part of GTVnx-I after radiotherapy was over 65 Gy. Our follow-up results showed that no recurrence occurred in the regression areas of GTVnx/rpn/nd-I which were delineated as CTV1/rpn1/nd1-II, and the 3-year survival rate was not decreased as compared with previously reported outcomes [9]. CTV2 was not prescribed any dose in plan-II, whereas a total dose of 45–47 Gy in 25–26 fractions was prescribed for CTV2 in plan-I. Historically, the suggested dose for microscopic sterilization was 45–50 Gy at 1.8–2 Gy/fraction [10]. Zhang et al. [11] analyzed prognostic factors of 1302 NPC patients based on a 10-year follow-up and found that the 5- and 10-year survival rates of patients without cervical lymph node metastasis who underwent 40–45 Gy irradiation were similar to those of patients with clinical adenopathy who underwent 50–60 Gy irradiation. The present study showed no recurrence in the CTV2 area. This outcome needs to be confirmed in long-term follow-up.

According to the principle of radiobiology, the tumor-killing effect of radiation is related to not only the fractionated dose but also the total dose. Wang et al. [2] and Yang et al. [3] used the same dose fractionation for each target volume in plan-II as that in plan-I, which may facilitate a simple superposition assessment of the doses between plans. Fung et al. [12] used the same dose fractionation, 2.1 Gy/fraction, for plan-I and plan-II over 7 weeks and used a higher dose fractionation, 3.5–3.7 Gy/fraction, for plan-III. The total dose for the three plans was as large as 80.9 Gy in 37 fractions or 84 Gy in 38 fractions, but the efficacy or toxic adverse effects of radiotherapy were not reported. The dose hyperfractionation in plan-II might increase the possibility of late reaction tissue damage and may elicit serious sequelae. In the present study, a higher dose per fraction was prescribed for GTVnx-II and CTV1-II with the intention of increasing the biological effect of radiation and improving therapeutic effect.

In conclusion, our adaptive replanning IMRT for patients with NPC provides a new perspective on target redelineation and dose prescription, as it would demonstrate a significant dosimetric and clinical benefits without recurrence and reduction in survival.

## Additional file

**Additional file 1: Table S1.** Changes in target volumes and volumes of OARs between plan-I and plan-II. **Table S2.** Relative doses for targets and OARs between plan-I and plan-II.

## Abbreviations

NPC: nasopharyngeal carcinoma
IMRT: intensity-modulated radiotherapy
GTV: gross tumor volume
nx: nasopharynx
nd: lymph node
rpn: retropharyngeal lymph node
R: right
L: left
CTV: clinical target volume
Dmean: mean dose
OARs: organs at risk
LRRFS: locoregional failure-free survival
DMFS: distant failure-free survival
OS: overall survival
RTOG: Radiation Therapy Oncology Group
