# Supplementary material for: Target delineation and dose prescription of adaptive replanning intensity-modulated radiotherapy for nasopharyngeal carcinoma
Source: Cancer Commun (Lond). 2019 Apr 15;39:18. doi: 10.1186/s40880-019-0364-x (PMC6466715; doi:10.1186/s40880-019-0364-x)
Supplement: Supplementary file 1 — Additional file 1: Table S1. Changes in target volumes and volumes of OARs between plan-I and plan-II. Table S2. Relative doses for targets and OARs between plan-I and plan-II. [file 40880_2019_364_MOESM1_ESM.doc]

Table S1 Changes in target volumes and volumes of OARs between plan-I and plan-II

| Target/OAR | Average volume (cm^3^) | | | *P* value |
| --- | --- | --- | --- | --- |
|  | Plan-I | Plan-II | Median change (%) |  |
| GTVnx | 14.2 | 10.9 | -25.1 | 0.032 |
| GTVrpn-L | 1.7 | 0.9 | -22.5 | 0.136 |
| GTVrpn-R | 2.1 | 1.3 | -25.0 | 0.130 |
| GTVnd-L | 6.3 | 3.3 | -32.8 | 0.056 |
| GTVnd-R | 10.8 | 6.5 | -38.2 | 0.045 |
| Parotid-L | 28.8 | 22.1 | -23.4 | <0.001 |
| Parotid-R | 28.5 | 22.0 | -21.9 | <0.001 |
| SMG-L | 8.6 | 6.6 | -23.4 | <0.001 |
| SMG-R | 8.7 | 6.8 | -23.0 | <0.001 |
| Thyroid | 16.8 | 16.1 | -3.6 | 0.409 |
| Hypopharynx | 11.9 | 12.2 | 0.0 | 0.866 |
| Oropharynx | 18.4 | 19.3 | 0.0 | 0.414 |
| Oral cavity | 148.6 | 145.3 | 0.0 | 0.768 |

OARs: organs at risk, GTV: gross tumor volume (nx: nasopharynx; nd: lymph node; rpn: retropharyngeal lymph node; R: right; L: left), SMG: submandibular gland.

Table S2. Relative doses for targets and OARs between plan-I and plan-II

| Target/OAR | Dmean% (%) | | *P* value |
| --- | --- | --- | --- |
|  | Plan I | Plan II |  |
| PGTVnx | 104.3 | 104.7 | 0.103 |
| PGTVrpn-L | 102.9 | 103.2 | 0.729 |
| PGTVrpn-R | 103.1 | 103.1 | 0.948 |
| PGTVnd-L | 100.3 | 103.0 | 0.013 |
| PGTVnd-R | 101.5 | 103.1 | 0.010 |
| Brain stem | 50.8 | 39.1 | <0.001 |
| Spinal cord | 46.8 | 26.1 | <0.001 |
| Optic chiasm | 57.5 | 18.1 | <0.001 |
| Pituitary | 77.0 | 55.3 | <0.001 |
| Oral cavity | 50.7 | 42.4 | <0.001 |
| Oropharynx | 72.3 | 62.1 | <0.001 |
| Hypopharynx | 65.8 | 49.3 | <0.001 |
| Thyroid gland | 64.0 | 3.1 | <0.001 |

OARs: organs at risk; Dmean%, the percentage of the mean dose in the total dose; PGTV, planning target volume (nx: nasopharynx; nd: lymph node; rpn: retropharyngeal lymph node; R: right; L: left).
